# Supplementary material for: Pharmacokinetic–Pharmacometabolomic Approach in Early-Phase Clinical Trials: A Way Forward for Targeted Therapy in Type 2 Diabetes
Source: Pharmaceutics. 2022 Jun 15;14(6):1268. doi: 10.3390/pharmaceutics14061268 (PMC9231303; doi:10.3390/pharmaceutics14061268)

Supplementary Information

Contents

Table S1. Representative LCMS vials sequence 2

Figure S1. Chromatogram for metformin 3

Figure S2. Internal standard boxplots 5

Figure S3. Representative EigenMS batch correction principal component analysis diagram for metformin 1000mg plasma (Dataset A) in positive mode 6

Table S2. Number of features in the data processing flow 7

Table S3. Glucose monitoring at pre-dose and 4 hours post-dose (before lunch) 9

Figure S4. Representative metformin calibration curve 10

Table S4. Method validation data 11

Table S5. Individual subject metformin plasma concentration and pharmacokinetic parameters 15

Table S5.1 Metformin plasma concentration for individual subjects 15

Table S5.2 Pharmacokinetic parameters for individual subjects 16

Figure S5. Boxplot of Metformin plasma and urine samples in positive mode 17

Table S1. Representative LCMS vials sequence

**LCMS vial sequence**
Blank
Blank with internal standards
Blank plasma
Blank plasma with internal standards
MetP-PQC1
MetP-PQC2
MetP-PQC3
MetP-PQC4
MetP-PQC5
MetP-PQC6
MetP-S1T0
MetP-S1T2.5
MetP-S1T3
MetP-S1T3.5
MetP-PQC7
MetP-S2T0
MetP-S2T2.5
MetP-S2T3
MetP-S2T3.5
MetP-PQC8
MetP-S3T0
MetP-S3T2.5
MetP-S3T3
MetP-S3T3.5
MetP-PQC9
MetP-S4T0

MetP-S4T2.5
MetP-S4T3
MetP-S4T3.5
MetP-PQC10
MetP-S5T0
MetP-S5T2.5
MetP-S5T3
MetP-S5T3.5
MetP-PQC11
MetP-S6T0
MetP-S6T2.5
MetP-S6T3
MetP-S6T3.5
MetP-PQC12

Figure S1. Chromatogram for metformin

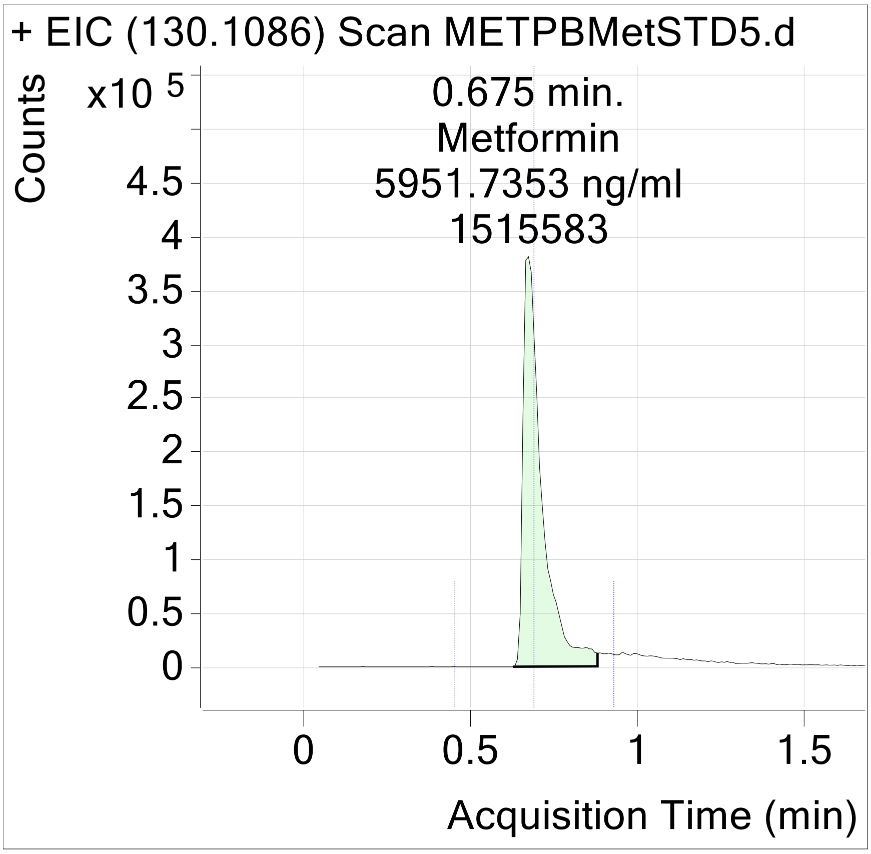
+ EIC (130.1086) Scan METPBMetSTD5.d
Counts
x10 5
0.675 min.
Metformin
5951.7353 ng/ml
1515583
4.5
4
3.5
3
2.5
2
1.5
1
0.5
0
0
0.5
1
1.5
Acquisition Time (min)

Figure S2. Internal standard boxplots

Internal standard gliclazide abundance boxplot Metformin 1000 mg in positive mode **(A)** and negative mode **(B)** for six raw spectral batches.

A

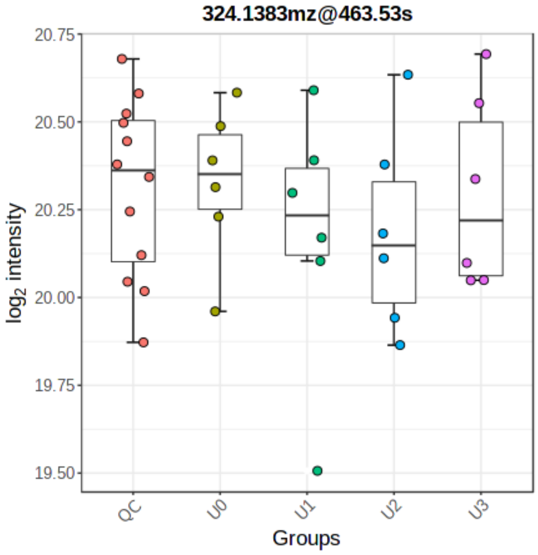

B

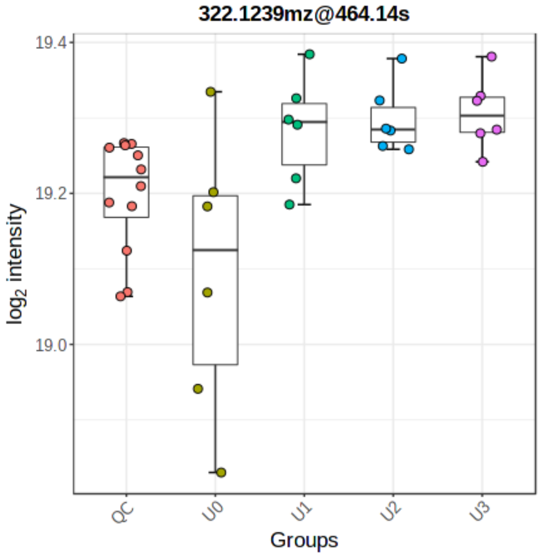

Figure S3. Representative EigenMS batch correction principal component analysis diagram for metformin 1000mg plasma (Dataset A) in positive mode.

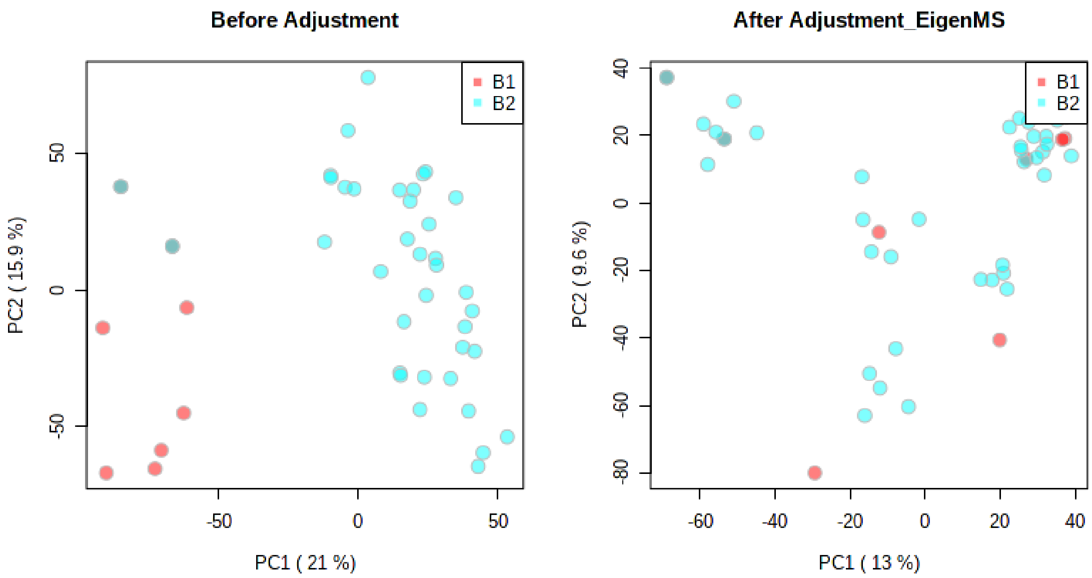

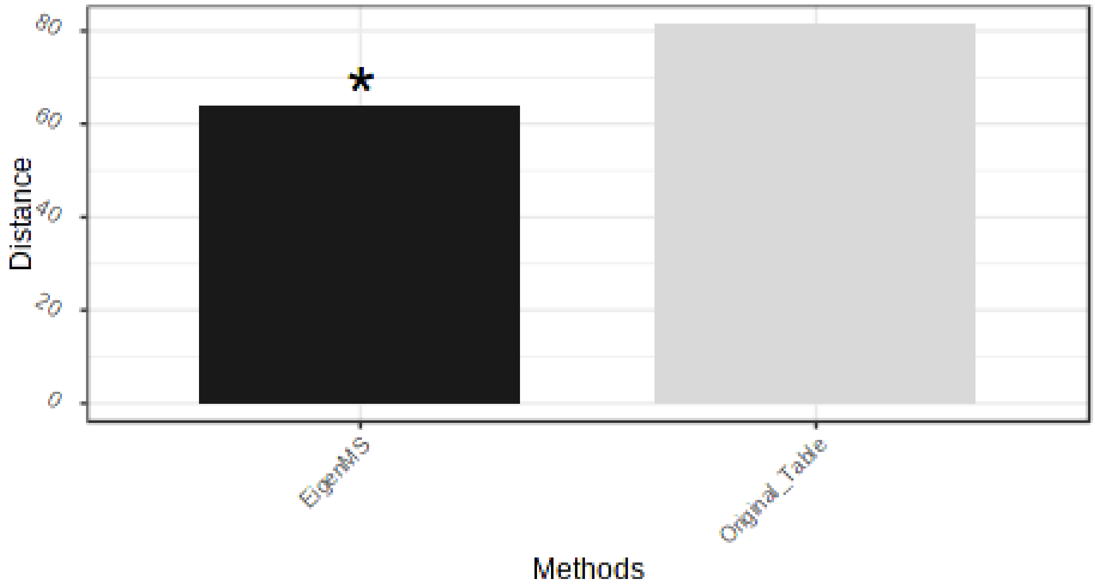

Table S2. Number of features in the data processing flow

Batch	ION	Algorithm	Normalization	Number of features (# samples) in MS Spectral processing	Number of features (# samples) in functional metaanalysis
Met Plasma Dataset A	Positive (LCMS-QTOF 1290 6550)	centWave, 15ppm	PQN, log10, range Scaling	9076 (45 samples)	T0 vs T2.5 – 2426 m/z (12 samples)<br>T0 vs T3 – 2433 m/z (12 samples)<br>T0 vs T3.5 – 2439 m/z (12 samples)
Metformin plasma Dataset A	Negative (LCMS-QTOF 1290 6550)	centWave, 15ppm	PQN, log10, range Scaling	3997 (43 samples)	T0 vs T2.5 – 3884 m/z (12 samples)<br>T0 vs T3 – 3898 m/z (12 samples)<br>T0 vs T3.5 – 3929 m/z (12 samples)
Metformin plasma Dataset B	Positive (LCMS-QTOF 1290 6530)	centWave, 15ppm	PQN, log10, range Scaling	2357 (27 samples)	Pre-Peak Dataset A – 2420 m/z (12 samples)<br>Pre-Peak Dataset B – 2349 m/z (22 samples)
Metformin plasma dataset B	Negative (LCMS-QTOF 1290 6530)	centWave, 15ppm	PQN, log10, range Scaling	895 (27 samples)	Pre-Peak Dataset A – 3888 m/z (12 samples)<br>Pre-Peak Dataset B – 894 m/z (22 samples)
Metformin urine	Positive (LCMS-QTOF 1290 6550)	centWave, 15ppm	PQN, log10, range Scaling	2016 (36 samples)	U0 vs U1 – 1996 m/z (12 samples)<br>U0 vs U2 – 2009 m/z (12 samples)<br>U0 vs U3 – 2012 m/z (12 samples)

Metformin urine	Negative (LCMS-QTOF 1290 6550)	**ce**ntWave, 15ppm	PQN, cubic root transformation, range scaling	2279 (36 samples)	U0 vs U1 – 2202 m/z (12 samples) U0 vs U2 – 2247 m/z (12 samples) U0 vs U3 – 2265 m/z (12 samples)

Table S3. Glucose monitoring at pre-dose and 4 hours post-dose (before lunch)

	Glucose (mmol/L)	
	Pre-dose	4 hours post-dose
S01	5.1	5.0
S02	5.2	4.7
S04	5.7	4.8
S05	4.8	5.2
S06	5.3	4.6
S07	5.2	4.7
S08	5.0	4.3
S09	4.7	4.4
S10	5.1	4.9
S11	4.7	4.7
S12	5.4	5.2
S13	5.5	4.4
S14	5.9	4.7
S15	5.1	4.4
S16	6.1	4.9
S17	4.8	4.6
S18	5.4	3.9
Mean	5.2	4.7

Figure S4. Representative metformin calibration curve

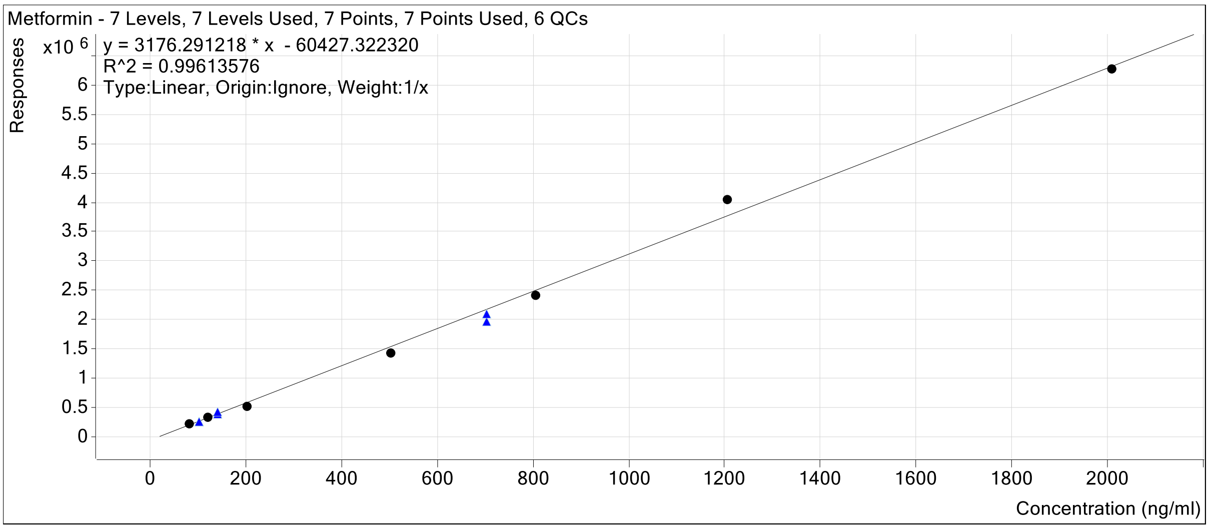

Table S4. Method validation data

S4.1 Results for accuracy and precision in three batches

	LLOQ 80.40 ng/mL	LQC 100.50 ng/mL	MQC 140.70 ng/mL	HQC 703.5 ng/mL
Accuracy and precision 1	89.01	101.39	144.73	628.07
	89.36	103.11	142.62	625.01
	88.98	101.66	140.84	626.25
	90.53	101.50	141.51	668.64
	89.03	101.86	152.58	641.60
Mean	89.38	101.90	144.45	637.92
Precision(%CV)	0.74	0.68	3.31	2.89
Accuracy (%)	111.17	101.40	102.67	90.68
n	5	5	5	5
Accuracy and precision 2	82.82	90.79	136.12	649.38
	79.11	94.39	136.01	629.36
	79.72	96.88	137.28	628.33
	79.75	91.63	135.97	630.00
	79.90	90.84	131.58	615.10
Mean	80.26	92.91	135.39	630.43
Precision(%CV)	1.82	2.87	1.62	1.94
Accuracy (%)	99.82	92.45	96.23	89.61
n	5	5	5	5
Accuracy and precision 3	90.85	98.60	147.63	747.62
	85.01	97.53	138.04	735.66
	88.77	93.31	139.24	699.98
	93.12	93.76	140.42	697.36
	81.02	90.70	144.84	694.07
Mean	87.76	94.78	142.04	714.94
Precision(%CV)	5.47	3.42	2.85	3.47
Accuracy (%)	109.15	94.31	100.95	101.63
n	5	5	5	5
Global statistic	LLOQ	LQC	MQC	HQC
Mean	85.80	96.53	140.63	661.10
Precision(%CV)	5.74	4.78	3.78	6.56
Accuracy (%)	106.71	96.05	99.95	93.97
n	15	15	15	15

LLOQ= Lower limit of quantitation, LQC = Low quality control, MQC = Medium quality control, HQC = High quality control

S4.2 Calibration Curve for three batches

	Cal1	Cal2	Cal3	Cal4	Cal5	Cal6	Cal7	R^2
Batch1	90.0	122.2	184.7	462.5	795.0	1205.0	2063.8	0.998
Batch2	92.7	106.5	185.0	467.4	809.0	1303.5	1953.9	0.995
Batch3	70.7	105.0	193.4	468.5	797.7	1203.6	2053.8	0.999
Mean	84.5	111.3	187.7	466.2	800.5	1237.4	2023.8	
Precision (%CV)	14.2	8.6	2.6	0.7	0.9	4.6	3.0	
Nominal value	80.4	120.6	201.0	502.5	804.0	1206.0	2010.0	
Accuracy (%)	105.1	92.3	93.4	92.8	99.6	102.6	100.7	

S4.3 Recovery results

Quality control	Unextracted area response	Recovery	Extracted area response
HQC (5025 ng/mL)	2195032		2071803
	2203199		2037689
	2210046		1935904
	2244972		1928429
	2230921		1919048
Mean	2216834		1978575
Recovery (%)		89.25	
MQC (703.5 ng/mL)	345916		360217
	364268		332851
	355210		336282
	345916		339653
	364268		352246
Mean	355115		344250
Recovery (%)		96.94	
LQC (301.5 ng/mL)	260163		220339
	268974		217278
	249786		205259
	271125		206527
	266625		197804
Mean	263334		209441
Recovery (%)		79.53	
Mean recovery		88.58	
Standard Deviation		8.72	

% Coefficient variation	**9.85**

S4.4 Carryover effects: Representative chromatograms for (a) blank plasma and (b) high quality control

(a)

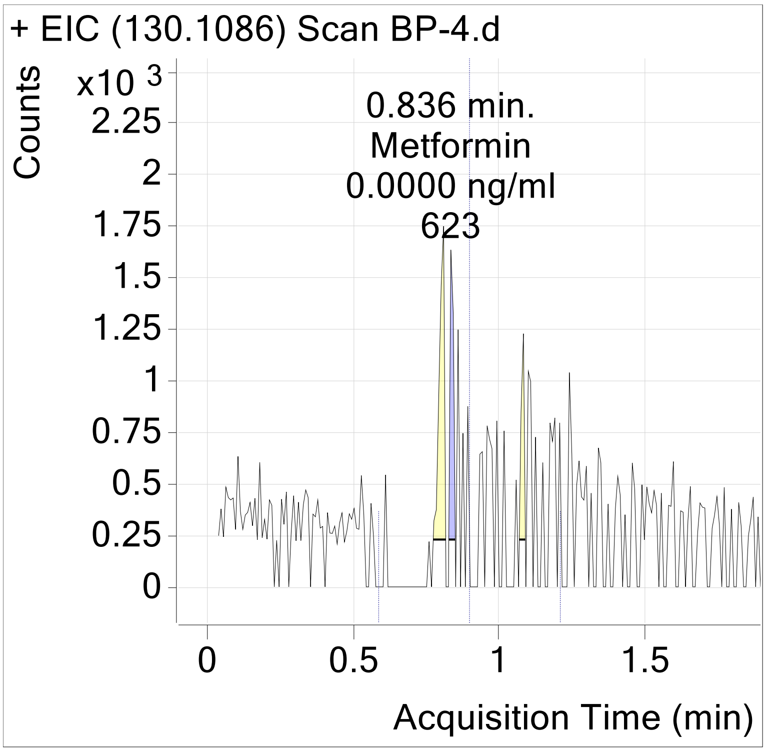

(b)

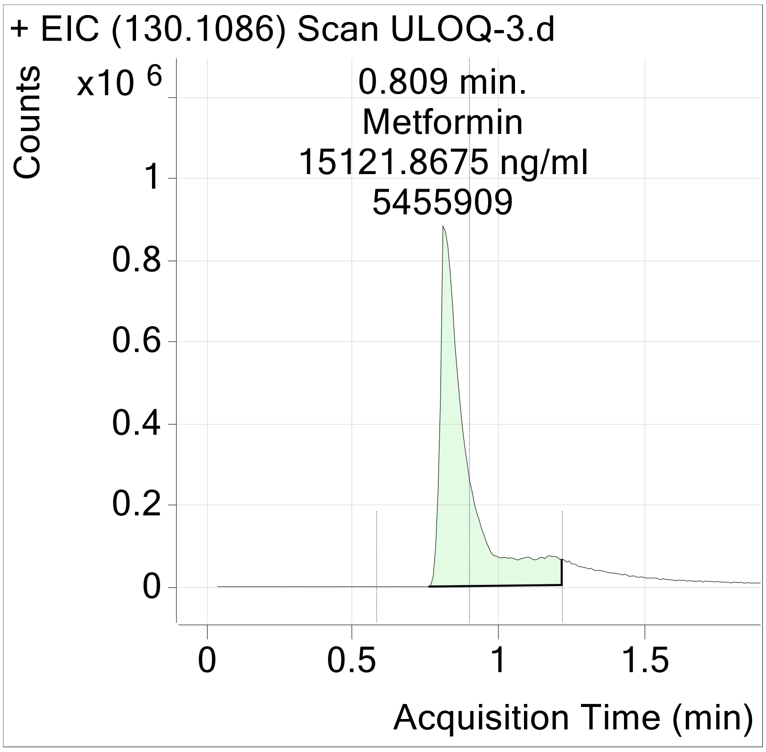
+ EIC (130.1086) Scan ULOQ-3.d
x10 6
Counts
0.809 min.
Metformin
15121.8675 ng/ml
5455909
1
0.8
0.6
0.4
0.2
0
0
0.5
1
1.5
Acquisition Time (min)

Table S5. Individual subject metformin plasma concentration and pharmacokinetic parameters

Table S5.1 Metformin plasma concentration for individual subjects

Time	Metformin plasma concentration (ng/ml)																
(hr)	S1	S2	S4	S5	S6	S7	S8	S9	S10	S11	S12	S13	S14	S15	S16	S17	S18
0	0.0	0.0	0.0	0.0	0.0	0.0	0.0	0.0	0.0	0.0	0.0	0.0	0.0	0.0	0.0	0.0	0.0
0.5	519.5	417.2	805.7	254.5	196.4	280.6	61.9	785.3	462.7	321.4	778.8	481.0	728.0	651.8	873.0	1076.3	1012.1
1	642.0	643.1	1374.5	507.3	570.2	1110.8	1072.3	1001.1	731.7	675.7	1032.4	783.7	761.7	726.7	1326.5	1206.1	1377.6
1.5	639.7	**654.4**	1346.3	572.8	589.2	**1335.5**	1079.4	1144.4	939.4	902.9	1214.1	931.1	918.1	758.8	1433.7	1229.7	1451.5
2	626.5	642.3	1389.1	686.6	**628.0**	1232.8	1168.2	1148.3	1139.7	914.8	1393.6	1112.5	993.1	765.8	**1652.5**	1171.6	1448.6
2.5	**700.2**	595.7	**1426.4**	772.6	457.6	1152.3	1122.3	**1172.5**	1245.8	**1151.8**	**1423.4**	1176.4	**1015.3**	766.7	1514.8	1067.0	**1557.2**
3	590.5	578.8	1422.9	**778.3**	426.1	1037.4	1170.9	1024.5	**1391.3**	999.2	1248.4	**1248.1**	970.5	807.6	1249.2	1191.7	1314.4
3.5	568.4	549.4	1416.6	714.5	404.3	960.9	**1255.2**	951.5	1370.7	1013.3	1155.9	1162.6	782.3	836.8	1141.9	1332.2	1186.3
4	504.8	540.4	1330.2	702.8	390.5	852.5	1088.2	859.6	1183.4	939.3	1030.2	1158.4	691.5	**849.5**	1078.6	**1381.7**	1136.6
5	390.3	524.9	1008.6	612.7	376.2	662.2	868.8	789.4	926.9	756.4	877.2	854.9	658.3	703.5	910.1	1215.9	900.9
6	297.6	395.0	561.0	407.1	235.5	498.9	598.7	577.9	708.1	704.3	627.0	577.1	516.8	507.4	602.1	961.6	626.4
8	200.8	282.2	434.0	279.3	168.5	355.4	404.2	323.4	443.1	492.0	355.6	410.1	282.0	288.6	400.2	577.6	457.5
10	139.7	204.4	238.4	194.1	140.8	203.1	269.9	221.5	287.3	333.4	262.0	273.8	208.1	261.0	237.0	471.5	298.8
12	106.4	138.5	157.1	122.0	101.2	141.7	173.4	167.4	222.8	235.5	183.0	176.8	139.0	151.1	176.7	258.1	224.1
24	38.1	43.7	38.0	43.2	30.6	26.1	57.6	53.3	80.4	75.5	69.1	56.5	51.7	30.7	59.5	75.0	72.5

(highlighted) Maximum plasma concentration

Table S5.2 Pharmacokinetic parameters for individual subjects

ID	Cmax (ng/mL)	Tmax (h)	Thalf (h)	$AUC_{0\text{-}t}$ (ng.h/mL)	$AUC_{0\text{-}\infty}$ (h.ng/mL)	CL (l/h)	Ke (h^{-1})
S1	700.2	2.5	7.7	5013.3	5435.7	0.184	0.090
S2	654.4	1.5	5.4	5768.2	6109.5	0.164	0.128
S4	1426.4	2.5	5.5	10111.1	10411.2	0.096	0.127
S5	778.3	3	6.9	5953.7	6384.8	0.157	0.100
S6	628.0	2	6.5	4169.1	4456.7	0.224	0.107
S7	1335.5	1.5	4.8	7870.0	8050.3	0.124	0.145
S8	1255.2	3.5	6.7	8955.5	9510.3	0.105	0.104
S9	1172.5	2.5	7.0	8496.0	9032.2	0.111	0.099
S10	1391.3	3	7.8	10020.1	10924.2	0.092	0.089
S11	1151.8	2.5	6.8	9259.1	9998.0	0.100	0.102
S12	1423.4	2.5	7.7	9644.7	10409.5	0.096	0.090
S13	1248.1	3	6.5	8981.7	9512.0	0.105	0.107
S14	1015.3	2.5	6.8	7299.6	7810.6	0.128	0.101
S15	849.5	4	4.8	7099.6	7313.6	0.137	0.143
S16	1652.5	2	7.2	10086.8	10707.4	0.093	0.096
S17	1381.7	4	4.7	12187.0	12697.0	0.079	0.147
S18	1557.2	2.5	7.0	10883.1	11618.5	0.086	0.099
Mean	1154.21	2.65	6.46	8341.1	8845.97	0.122	0.110
SD	326.32	0.724	01.03	2197.3	2317.45	0.039	0.020

Figure S5. Boxplot of Metformin plasma and urine samples in positive mode

1. Boxplot for metformin plasma samples in positive mode (Dataset A)

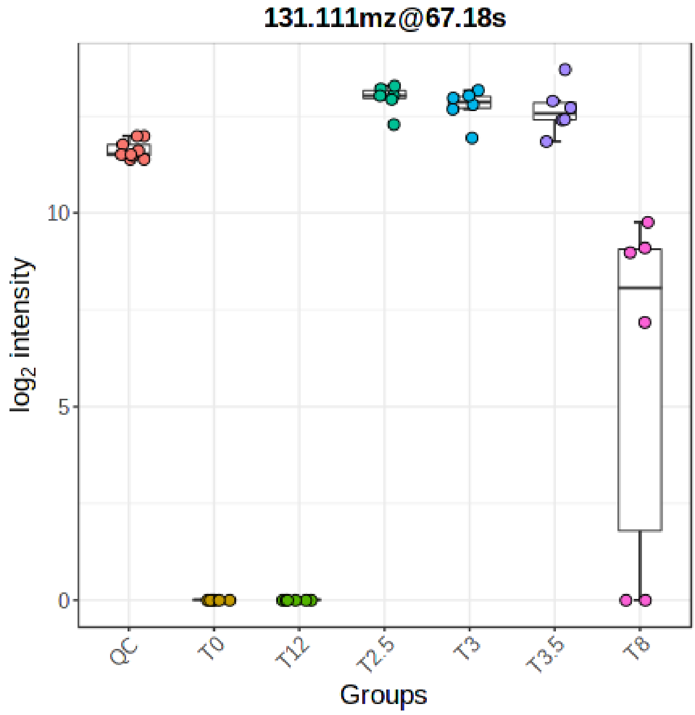

2. Boxplot for metformin urine samples in positive mode

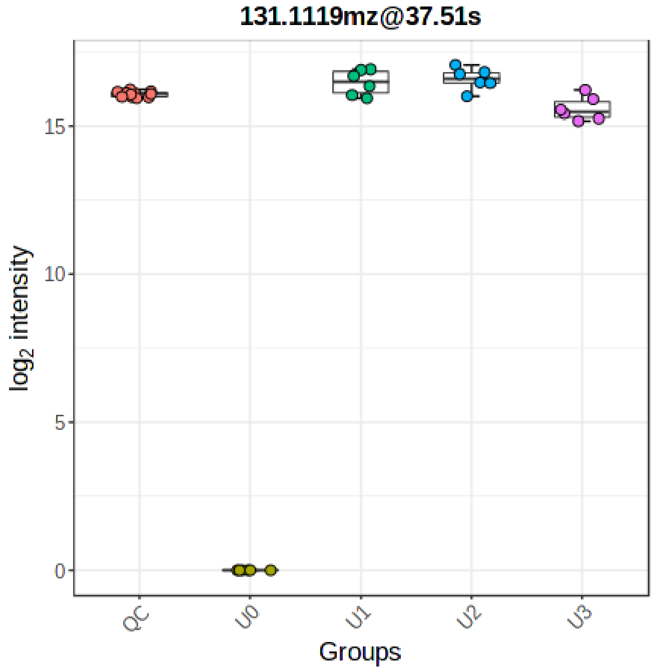

Supplement: Supplementary file 1 [file pharmaceutics-14-01268-s001.zip › pharmaceutics-1731469-supplementary.pdf]
